# Prospective evaluation of computer-assisted analysis of skeletal lesions for the staging of prostate cancer

**DOI:** 10.1186/s12880-017-0211-y

**Published:** 2017-07-10

**Authors:** Lars J. Petersen, Jesper C. Mortensen, Henrik Bertelsen, Helle D. Zacho

**Affiliations:** 1 Department of Nuclear Medicine, Clinical Cancer Research Center, Aalborg University Hospital, Hobrovej 18-22, DK-9000 Aalborg, Denmark; 2 Department of Clinical Medicine, Aalborg University, Aalborg, Denmark; 3 Department of Nuclear Medicine, Regional Hospital West Jutland, Herning Gl. Landevej 61, DK-7400 Herning, Denmark; 4 Department of Clinical Physiology and Nuclear Medicine, Randers Hospital, Randers, Denmark; 5 Department of Clinical Physiology, Viborg Hospital, Viborg, Denmark

**Keywords:** Agreement, Bone scan index, Bone scintigraphy, Computer-aided analysis, Diagnostic properties, Predictive values, Prostate cancer

## Abstract

**Background:**

The purpose of this study was to compare the agreement of the bone scan index (BSI) using EXINI Bone^BSI^ versus experts’ readings in the initial staging for bone metastasis in prostate cancer. In addition, the diagnostic outcome was assessed in a large subset of patients where a true reference for metastases could be determined based on clinical and biochemical follow-up and/or supplementary imaging.

**Methods:**

A total of 342 patients had a bone scintigraphy as part of routine staging for prostate cancer. Supplementary imaging was obtained at the discretion of the referring urologist. After full recruitment, the BSI and the number of malignant lesions were calculated using EXINI Bone^BSI^, and three imaging experts independently classified bone status by a dichotomous outcome (M1 for bone metastasis, M0 for no bone metastasis). A true reference was available in a subset of the patients based on post-operative prostate-specific antigen responses after radical prostatectomy and/or supplementary imaging.

**Results:**

Software analysis with a BSI > 0 as the cut-off for metastasis showed excellent agreement with expert classification for M1 disease (96% of the patients) but modest agreement for M0 disease (38%). With a BSI > 1, the agreement was 58% for M1 and 98% for M0. Software analyses based on individual European Association of Urology risk classification did not improve the diagnostic performance. Among patients with a true reference, the software showed metastasis in 64% of the M0 patients but correctly classified metastases in all M1 patients. The sensitivity was 100%, the specificity was 36%, the positive predictive value was 12.6% and the negative predictive value was 100% with a BSI >0 compared with 66.7%, 97.8%, 72.7%, and 97.0% with a BSI > 1.

**Conclusion:**

The diagnostic value of using EXINI Bone for the BSI in the staging of newly diagnosed prostate cancer is limited.

## Background

Planar bone scintigraphy (BS) has been recommended for the assessment of bone metastasis in newly diagnosed prostate cancer for decades [1, 2]. The reading of BS can be a timely task, and it may be biased by the extent and nature of the clinical data, the access to prior imaging results, and the experience of the readers [3]. Computer-aided analysis is an objective method, which may improve the classification of M status and thus optimize patient management. EXINI Bone^BSI^ is a Food and Drug Administration-approved software for the analysis of planar BS with solid technical and clinical documentation [4–10].

The bone scan index (BSI), the cumulated tumour load of malignant lesions in the entire skeleton are the most used endpoints with this software, which has been assessed in a number of clinical scenarios. The BSI has been shown to carry solid prognostic value for estimating the outcome and survival in metastatic, hormone-naïve and castration-resistant prostate cancer [9–15] and to be valuable in the assessment of treatment response to anti-cancer treatments [11, 16, 17]. The diagnostic value of the BSI for the staging of newly diagnosed prostate cancer has not been assessed so far.

The purpose of this study was to compare the diagnostic value of the BSI versus a panel of trained nuclear medicine experts for the classification of metastatic disease in a large prospective cohort of patients with newly diagnosed prostate cancer.

## Methods

### Study design and patients

Data for this study were collected from a large prospective study of bone imaging in newly diagnosed prostate cancer. All patients had a BS conducted for staging purposes as a part of institutional practice. Consecutive prostate cancer patients were recruited from three sites for this non-interventional study in Central Region Jutland, Denmark, from March 2008 to October 2009 [18]. Due to the design, a true reference for M0/M1 status was not available in all patients.

### Bone scintigraphy and supplementary imaging

Whole body, planar ^99m^Tc-bone scintigraphy was obtained as previously described [18]. The BS report was described according to institutional practice (involving at least one specialist at each site). The need for additional single photon emission computed tomography/computed tomography (SPECT/CT) was determined by the nuclear medicine physician in charge, whereas the use of supplementary magnetic resonance imaging (MRI) and/or CT was at the discretion of the referring urologist. The original study population consisted of 635 patients [18], of which 614 patients had a whole-body BS suitable for software analysis [19]. Data from one site (n = 272 patients) was excluded in this report due to the use of Interfile format for data storage. It has previously been shown that EXINI Bone^BSI^ provided significantly different results for Digital Imaging and Communications in Medicine (DICOM) file formats, compared to Interfile file formats [19]. Thus, this report included data for 342 patients from two sites that used DICOM files, which is the most common file format and the only format that is allowed in subsequent versions of the software.

### Expert reading of bone scintigraphy

All BS images were retrospectively read independently by three board-certified nuclear medicine physicians under standardized conditions. No clinical, laboratory, or pathology data were available during the BS reading. The three experts unanimously agreed on the M1/M0 classification in 96% of the cases [20]. In the case of disagreement, majority voting was used.

### Computer-assisted analysis of bone scintigraphy

We tested EXINI Bone^BSI^ version 1.6.2 (EXINI Diagnostics AB, Lund Sweden). Company representatives ensured correct installation and use of the software at all sites. EXINI Bone^BSI^ is a computer-assisted software that can predict the malignancy on bone scans based on a patented algorithm. The software identifies the skeletal site of pathological uptake and classifies these sites as benign (cold spots) or malignant (hot spots), as shown graphically by Sadik et al. [5]. The segmentation is also accessible at http://bonescanindex.org/about-bsi/. The BSI is derived from whole-body bone scans in which the skeleton is outlined and segmented into regions of interest. The contours of all the hot spots are calculated and bone tumour involvement in each region is automatically calculated as the proportional area of the involvement, adjusted for differences in the skeletal mass between regions.

### Patient-specific risk assessment

EXINI Bone^BSI^ provides the option to select settings in terms of the probability of bone metastasis, i.e., ‘balanced mode’, ‘high specificity’ and ‘high sensitivity’. We used ‘balanced mode’ as the default. The individual risk was calculated based on a modified European Association of Urology (EAU) risk classification [2], as previously described [19]. Thus, patients were classified as follows: 1) low risk if T1-T2, Gleason 2–6, and prostate specific antigen (PSA) < 10 ng/mL; 2) intermediate risk if T1-T2, Gleason 7 or PSA 10–20 ng/mL; 3) high risk if T3 or Gleason score 8–10 or PSA >20 ng/mL; or 4) very high risk if T4 or any T, N1. For patient-specific setting analyses, the data from patients with low risk prostate cancer were analysed using the ‘high specificity’ setting, data from patients with high or very high risk were analysed using the ‘high sensitivity’ setting, and data from patients with intermediate risk were analysed using the ‘balanced’ mode analysis.

### Patients with true M0 or M1 disease

Additional analyses were performed in patients who were defined as true M0 or M1 based on additional imaging and/or clinical follow up. A number of patients underwent radical prostatectomy and presented with PSA < 0.1 ng/mL within 6–12 months after surgery (*n* = 62). This group was classified as true M0 [21]. An additional 68 patients were classified with benign or equivocal findings in the clinical BS report and were confirmed as non-metastatic by supplementary MR, CT, and/or SPECT/low-dose CT. The total M0 population thus comprised 130 patients. Another group of patients was classified by MR or CT as true M1 based on a likely or definitive malignant BS with confirmed malignancy (*n* = 12).

### Statistics

The statistical tests used were t-tests with logarithmically transformed data, if required, using Stata 13 (StataCorp LP, Texas, USA). Agreements among the software and the readers were calculated, since the expert classification could not be regarded as a true reference. Diagnostic characteristics were calculated in patients with a true reference.

## Results

### Clinical data

A total of 342 patients were included in the final analysis. Their mean age was 70 ± 8 years, the median PSA was 19.9 ng/mL (range 1.5 -15,357), the median Gleason score was 7 (range 4–10), and the median T-stage was 2 (range 1 – 4). The vast majority of the patients (78%) had limited local disease (T1 or T2). The experts declared M1 status in 50 patients (14.6%).

### Correlation of software endpoints with expert reading

A manual correction of artefacts, e.g., lesions such as bladder catheters or bone implants that were classified as malignant lesions, was performed prior to analysis [19]. The BSI values, as well as the total number of malignant lesions, were significantly higher in patients who were classified as M1 by experts versus patients who were classified as M0 (both, *p* < 0.00001, Table 1). Among all the patients who were declared M0 by experts, approximately 40% had a BSI of zero, whereas the majority of the patients had a BSI > 0, and some patients had quite high BSI values (Table 1). For the 50 patients classified as M1, most patients had a BSI > 0.

**Table 1:** Data (the BSI and the numbers of hot spots) generated by EXINI Bone^BSI^ in the entire patient population as well as per M status as classified by blinded expert reading

Software variable	Total population	Expert classification	
	All (*n* = 342)	M0 (*n* = 292)	M1 (*n* = 50)
BSI			
Mean ± SD	0.653 ± 1.930	0.141 ± 0.233	3.650 ± 3.858
Median (range)	0.079 (0–12.280)	0.057 (0.0–2.004)	2.254 (0.0–12.280)
Number of hot spots			
Mean ± SD	6.8 ± 18.0	2.0 ± 3.1	35.1 ± 35.1
Median (range)	1 (0–127)	1 (0–21)	21 (0–127)

*BSI* bone scan index, *BS* bone scan, *M0* no metastasis, *M1* metastasis, *SD* standard deviation

### Agreement of diagnostic classification

No standardized diagnostic criteria for the detection of malignancy by a BSI have been published. However, a BSI > 0 can be considered to be the minimum malignant involvement of the skeleton [8, 11]. A BSI value of > 1 has been proposed as a limit to differentiate between impaired outcomes in prostate cancer patients without apparent metastasis [11] or minimal malignant bone involvement [8].

With a BSI > 0 as a cut-off, the software disagreed with the experts’ ratings of M0 for more than 60% of the patients (Table 2). The software generally agreed on the M1 status reported by the experts. In contrast, with a BSI > 1 as a cut-off, the software disagreed with the experts’ classification of M1 in 58% of the cases but showed notable agreement on the M0 classification.

**Table 2:** Agreement between EXINI Bone^BSI^ and expert reading for classification of metastasis

Software cut-off	Software classification	Expert classification	
		M0 (*n* = 292)	M1 (*n* = 50)
BSI > 0	M0	110 (37.7%)	2 (4.0%)
	M1	182 (62.3%)	48 (96.0%)
BSI > 1	M0	287 (98.3%)	21 (42.0%)
	M1	5 (1.7%)	29 (58.0%)

*BSI* bone scan index, *BS* bone scan, *M0* no metastasis, *M1* metastasis

### Patient-specific settings

A total of 336 of 342 patients had complete data sets for EAU risk classification. The use of patient-specific settings did not improve the agreement between the software and the experts. With a BSI > 0 as a cut-off, the software agreed with the experts on the M0 classification in only 33.1% of the patients, whereas the software classified M1 disease in 66.9% of the patients who were declared as M0 by experts. The software agreed with the experts on the M1 diagnosis in 95.9% of the patients. With a BSI > 1 as a cut-off, the software agreed on the M1 diagnosis by the experts in 57.1% of the cases and on the experts’ M0 diagnosis in 97.9% of the patients. These data are very similar to the data shown in Table 2.

### Patients with true M0 or M1 disease

The total true M0 population was comprised of 130 patients. The mean BSI was 0.156 (median 0.064, range 0 – 2.004). The mean number of hot spots was 2 (median 1, range 0 – 19). EXINI Bone^BSI^ detected at least one malignant lesion in 83 (64%) of these patients. Thirty of the 83 patients had one malignant lesion, 38 had 1 – 5 lesions, 11 had 6 – 10 lesions, and 4 had >10 lesions. Twelve patients were classified as having true M1 disease. These patients had at least one malignant lesion and thus a BSI > 0. The mean number of lesions was 40 (median 34, range 3 – 87). The mean BSI was 4.868 (median 3.318, range 0.304 - 12.280). The diagnostic characteristics of EXINI Bone^BSI^ versus patients with a true reference showed low specificity and a positive predictive value with a BSI > 0 and low sensitivity with a BSI > 1 (Table 3). The sensitivity and specificity presented in Table 3 are very similar to the agreement presented in Table 2, considering the ratings by the experts as the reference. The proportion of patients classified as M1 by the software versus M1 patients classified by blinded experts (‘sensitivity’) was 96.0% (48 of 50 patients) and 58.0% (29 of 50 patients) with a BSI of 0 and 1, respectively (Table 2). Accordingly, the corresponding ‘specificity’ was 37.7% and 98.3% with a BSI of 0 versus a BSI of 1, respectively. The wording ‘sensitivity’ and ‘specificity’ are used since expert ratings are not true references in diagnostic terminology. However, the calculations may improve the comparison between the data that are presented in Tables 2 and 3.

**Table 3:** Diagnostic properties of the BSI calculated by EXINI Bone^BSI^ in 142 patients with a true reference

EXINI Bone^BSI^		Sensitivity (%)	Specificity (%)	PPV (%)	NPV (%)
Metastasis present	Metastasis absent				
BSI > 0	BSI = 0	100%	36.2	12.6	100
BSI > 1	BSI ≤ 1	66.7	97.8	72.7	97.0

*BSI* bone scan index, *NPV* negative predictive value, *PPV* positive predictive value

## Discussion

A large number of publications have reported on the use of the computer-aided analysis of planar bone scans [22–24]. Commercially available software, such as EXINI Bone^BSI^, has become an established modality in both clinical settings and clinical trials for the objective and quantitative assessment of bone involvement in prostate cancer patients [4–8]. The clinical value of lesional data, in particular the BSI, has been investigated in many trials in different clinical scenarios, including in the prognosis of prostate cancer [11–15] and in the assessment of the treatment response to anti-cancer treatments [11, 16, 25]. To the best of our knowledge, no previous reports have focused on the diagnostic role of the BSI in the staging of newly diagnosed prostate cancer. The key findings of our study were that EXINI Bone^BSI^ declared M1 disease on a large proportion of patients who were classified by experts as M0 or missed M1 disease, depending on the BSI cut-off values. These findings were confirmed in patients with a true reference.

Different BSI values have been proposed to indicate metastatic disease. A BSI > 0 has been proposed by several groups as an indicator of malignant involvement of the skeleton [8, 12]. A BSI value of > 1 has been proposed as a limit to differentiate between impaired outcomes in prostate cancer patients without apparent metastasis [11], minimal malignant bone involvement [8], and an indicator of poor prognosis [13]. We observed that a BSI > 0 correctly identified M1 patients who were identified by experts but classified a large proportion of M0 patients with metastasis. In contrast, a BSI > 1 only identified bone metastasis in approximately 60% of the patients who were diagnosed as having M1 disease by experts. Thus, neither cut-off value is applicable in clinical practice.

Even though BS is used in clinical medicine to classify the presence or absence of metastatic disease, consensus or expert reading is not a true reference test. We applied supplementary imaging and/or the biochemical response after radical prostatectomy to reach a true reference. A large proportion of patients could not be classified as true M0 or M1 due to confounding variables that interfered with correct classification at the time of the BS. This includes an androgen deprivation therapy, radiation therapy with androgen blockade, watchful waiting/active surveillance, and a lack of confirmative imaging. This is a drawback of the non-interventional design, but it reflects daily practice. Still, among more than 140 patients with a true reference, we found a low specificity with a BSI > 0 and a low sensitivity with a BSI > 1, similar to the findings of agreement with the experts.

The findings of several malignant lesions in patients who were classified as M0 by experts or true M0 may raise concern regarding the use of hot spot analysis for the assessment of treatment response in metastatic prostate cancer. The number of new lesions is a key factor among the Prostate Cancer Working Group criteria for disease progression in metastatic, castration-resistant prostate cancer [26]. Kaboteh et al. used EXINI Bone^BSI^ to calculate new lesions and BSI values in patients who were treated with chemotherapy [27]. In comparison to an increase in the BSI, the appearance of two new lesions shown with EXINI Bone^BSI^ did not correlate with overall survival. We consider it premature to use the number of malignant lesions from computer-assisted analysis in clinical decision-making based on the existing documentation.

The tested version of the software came with options to adjust settings based on the pre-test risk of metastasis. Patient-specific settings, based on EAU risk classification, did not improve the diagnostic performance of the software. These findings are in line with our recent study on the use of a patient-specific setting for the diagnostic properties of EXINI Bone^BSI^ on a patient level [19]. This function has been removed in recent versions of the software.

The endpoints of this report, the analyses of lesional data, were different from the patient classification outcomes reported previously for the same population [19]. Two issues favour publication of the lesional data in addition to the patient classification outcomes that were previously reported: first, lesional analyses, in particular the BSI, are the most frequently reported outcomes in studies with EXINI Bone^BSI^; second, no clear association exists for the lesional analysis and patient classification. A total of 231 of 342 patients in this study were classified as definitive non-malignant (‘normal’) by software on a patient outcome classification, but 127 of these 231 ‘normal’ patients had at least one malignant lesion (and thus a BSI > 0). Sixty-seven patients had only one malignant lesion, 60 patients had more than one lesion, and four patients had 10 or more lesions. Thus, we find it relevant to report our data with the lesional data reported separately [28].

We manually corrected for artefacts, which the software wrongly classified as malignant lesions in approximately 15% of the cases. Therefore, the presented results represent a combination of software capabilities plus manual corrections. Without the adjustment of artefacts, many more lesions were malignant and the BSI values would be higher, likely resulting in more misclassifications of M0 patients. Finally, it should be emphasized that the present findings are valid for DICOM file data only; it has previously been shown that the diagnostic performance of EXINI Bone^BSI^ varied with the file format [19].

## Conclusions

This study investigated the performance of EXINI Bone^BSI^ on the lesional analysis in an unselected population of patients with newly diagnosed prostate cancer. Based on our observations, we do not find EXINI Bone^BSI^ suitable for staging in clinical practice.

## Abbreviations

BS: Bone scintigraphy
BSI: Bone scan index
CT: Computed tomography
DICOM: Digital Imaging and Communications in Medicine
EAU: European Association of Urology
M0: Bone metastasis not present
M1: Bone metastasis present
MRI: Magnetic resonance imaging
PSA: Prostate specific antigen
SPECT: Single photon emission computed tomography
